# Seroprevalence of Malaria and Hepatitis B Coinfection among Pregnant Women in Tamale Metropolis of Ghana: A Cross-Sectional Study

**DOI:** 10.1155/2018/5610981

**Published:** 2018-09-24

**Authors:** Gideon Kofi Helegbe, Paul Armah Aryee, Baba Sulemana Mohammed, Anthony Wemakor, David Kolbila, Abdul-Wahid Abubakari, Salam Askanda, Rashid Alhassan, Collins Barnie, Afua Aboagyewaa Donkoh, Ernest Ofosu

**Affiliations:** ^1^Department of Biochemistry and Molecular Medicine, School of Medicine and Health Sciences (SMHS), University for Development Studies (UDS), Tamale, Ghana; ^2^Department of Nutritional Sciences, School of Allied Health Sciences (SAHS), University for Development Studies (UDS), Tamale, Ghana; ^3^Department of Pharmacology, School of Medicine and Health Sciences (SMHS), University for Development Studies (UDS), Tamale, Ghana; ^4^Department of Obstetrics and Gynaecology, School of Medicine and Health Sciences (SMHS), University for Development Studies (UDS), Tamale, Ghana; ^5^Department of Nursing, School of Allied Health Sciences (SAHS), University for Development Studies (UDS), Tamale, Ghana

## Abstract

**Background:**

Coinfections are becoming common risk factors that may contribute to the increased burden of morbidity in pregnancy. The aim of this study was to assess the seroprevalence of coinfections of malaria, hepatitis B (HBV), human immunodeficiency virus (HIV), and syphilis among pregnant women attending antenatal clinics (ANC) in the Tamale Metropolis.

**Methods:**

By means of rapid diagnostic tests (RDTs), pregnant women attending the Tamale Teaching Hospital (TTH) were screened for malaria, HBV infection, HIV infection, and syphilis from March 2013 to February 2015. Haemoglobin (Hb) values, sickling, and glucose-6-phosphate dehydrogenase deficiency (G6PDd) statuses were also assessed using full blood count (FBC), sodium metabisulphite, and methaemoglobin reduction tests, respectively. Logistic regression analysis was performed to estimate the risks/odds ratios (ORs) for the coinfections and other variables (age, gravidity, and time of the first ANC visit) with 95% confidence intervals (CIs) and set *p* values for accepting any differences at <0.05.

**Results:**

Within the two-year study period, data were collected from 3,127 pregnant women. The mean age (SD) of the pregnant women was 28.5 (±5.0) years. Of the total number, seroprevalence was high for malaria (11.6%) and HBV infection (4.2%) and low for HIV infection (1.0%) and syphilis (0.4%) monoinfections. Mal/HBV coinfection was higher (0.7%) when compared with Mal/HIV (0.1%), Mal/syphilis (0.0%), HBV/HIV (0.0%), HBV/syphilis (0.1%), and HIV/syphilis (0.0%) coinfections. The mean Hb (g/dl) for the women with the four monoinfections was significantly different from one another (*p*=0.009). Pregnant women with malaria infection were about 2 times more likely to be coinfected with HBV even after adjusting for potential confounders (adjusted odds ratio (AOR) = 1.66, 95% CI = 1.04–2.65, *p*=0.031). Those in their third trimester and visiting the ANC for the first time were significantly less likely to be infected with HBV (AOR = 0.45, 95% CI = 0.28–0.73, *p*=0.001), with malaria/HBV coinfection (AOR = 0.09, 95% CI = 0.01–0.68, *p*=0.020), and with any coinfection (AOR = 0.19, 95% CI = 0.06–0.63, *p*=0.007).

**Conclusion:**

A comparatively high seroprevalence of malaria and its coinfection with HBV in pregnant women was observed in this study. Considering the effects that both malaria and HBV have on the liver, it would be expedient to conduct further studies to assess liver function among malaria/HBV-infected individuals, while interventions to prevent coinfections among pregnant women are intensified.

## 1. Background

Infectious diseases continue to remain life-threatening and a major public health problem globally. Children under 5 years and pregnant women remain most vulnerable to infectious diseases. While monoinfections have shown to be significant risk factors for poor pregnancy outcomes (e.g., low birth weight, anaemia, early foetal loss, stillbirth, and prematurity) among pregnant women [1, 2], studies that evaluated the impact of coinfections on the health of pregnant women [3–5] have come out with some relevant findings, albeit limited by relatively smaller sample sizes. Most of these studies have focused on soil-transmitted helminth (STH) and malaria [4, 6], TB [5], and human immunodeficiency virus (HIV) [7]. While malaria, HBV, and HIV infections are among the topmost diseases affecting many Ghanaians [8], including these pregnant women, syphilis infection among pregnant women is showing a rather disturbing global trend with concomitant adverse pregnancy outcomes [9, 10].

Malaria is well endemic in Ghana and has remained a public health problem in pregnancy. Among pregnant women, it accounts for 28.1% of out-patient department (OPD) attendance, 13.7% of admissions, and 9.0% of deaths [11]. Generally, persons living in areas of high transmission are known to acquire immunity to the disease over time [12]. However, in pregnancy, a suppression of the immune system rather occurs [13, 14], especially for primigravidae who tend to have a higher risk of malarial infection. Malaria in the pregnant woman, even when asymptomatic, can be serious and a major contributor to low neonatal birth weight, maternal anaemia, infant mortality, spontaneous abortion, and stillbirths [6, 15]. Coinfections of malaria with other infectious diseases have also been shown to have other outcomes. For instance, individuals coinfected with *Plasmodium* sp. and HBV present with lower parasitaemia and higher viremia [16]. This is because malaria infection is associated with strong CD4^+^ cell activation and upregulation of proinflammatory cytokines, which provides an ideal microenvironment for the spread of the HIV-1 among the CD4^+^ cells and thus for rapid viral replication [17]. Malaria/HIV coinfection has been shown to have the following effects: low Hb [18–20], low CD4 count in the first trimester [21], and low birth weight, fever, and urinary tract infection [20] among others.

The HBV monoinfection is a serious and common infectious disease of the liver. The World Health Organization (WHO) in 2009 reported HBV to infect nearly 2 billion people around the globe, including pregnant women. Activities such as unprotected sex, blood transfusion, tattoo, and sharing unprotected needles and blades predispose individuals to HBV including pregnant women [22]. In view of its potential deleterious effects on maternal and neonatal outcomes, the need for pregnant women to know their status much earlier is achieved by routine screening provided at the ANC. While some studies have documented high HBV infection prevalence (>8%) in Ghana [23, 24], data on its prevalence among pregnant women are scanty [25, 26]. Seroprevalence of HBV (8.03%) and HIV (17.3%) has been reported in a study in pregnant women in Tanzania [27]. Even though the mode of transmission of HBV is similar to that of HIV, it is 50 to 100 times more infectious [21]. A study among pregnant women indicates that 3.1% of HIV/HBV-seropositive women were HBeAg [28], where significantly higher HBV DNA load was also observed in these HIV/HBV-coinfected women. Coinfection of HBV/syphilis is, however, not common.

Infection with HIV for some decades has remained a major public health concern, especially in pregnant women [29, 30]. In the eastern region of Ghana, an overall HIV-positive prevalence of 8% has been shown among pregnant women [1]. The significant association between HIV infection and HBsAg-positive status and older age (>35 years) was also reported. This situation results in low CD4 count, which lowers immune status and leaves the affected individual immunocompromised. In view of that, individuals are prone to opportunistic infections such as TB [30] and HBV infections.

With respect to syphilis infection, over two million pregnancies worldwide are affected annually, resulting in adverse pregnancy outcomes and severe sequelae in the newborn [9, 10]. As part of the expanded programme on immunisation (EPI), Ghana's Health Policy recommends antenatal syphilis screening and treatment of positive clients to prevent vertical transmission [31]. Unfortunately, data available are scanty to further influence policy direction. The few studies that exist report 5.5% prevalence [10] using the point-of-care (POC) test. However, syphilis coinfection with other infections in pregnant women is not common.

Taken together, the impact of these major infectious diseases briefly reviewed here could significantly influence the health and well-being of pregnant women and their foetuses as either mono- or coinfections. Nevertheless, the literature on the coinfection seroprevalence of malaria, HBV, HIV, and syphilis among pregnant women in Ghana is lacking. Therefore, the objective of this study was to investigate the seroprevalence of coinfections of these four infectious diseases among pregnant women in Tamale.

## 2. Methodology

### 2.1. Study Area

**Figure 1:**
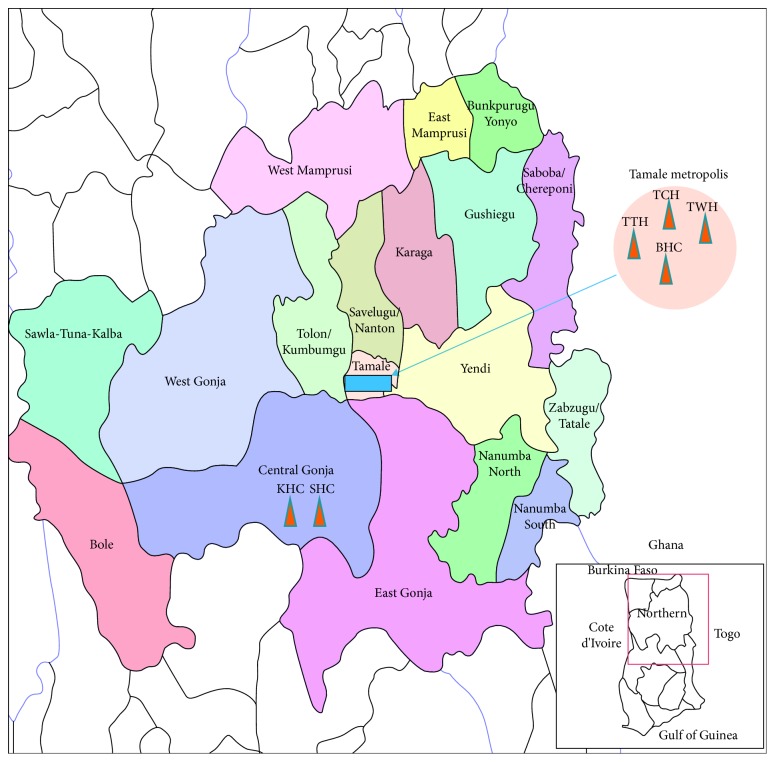
Map of Ghana showing the study area Tamale and neighbouring communities and districts. TTH: Tamale Teaching Hospital; TCH: Tamale Central Hospital; TWH: Tamale West Hospital; BHC: Bilpela Health Centre. These are examples of hospitals and health centers that serve the health needs of Tamale Metropolis and the environs, with TTH as the main referral Hospital.

The study was conducted in the Tamale Metropolis (Figure 1), specifically at the ANC of the Tamale Teaching Hospital (TTH), which is a tertiary referral hospital that provides health care services to the residents of Tamale Metropolis and all of northern Ghana as well as the neighbouring countries of La Cote d'Ivoire, Burkina Faso, and Togo. The hospital is located in the eastern part of the metropolis. The TTH was chosen because of its location, making it accessible to patients far and near.

### 2.2. Study Design

A hospital-based cross-sectional study design was employed to assess the seroprevalence of mono- and coinfections of 4 different infectious diseases affecting pregnant women attending ANC in the TTH from March 2013 to February 2015. The study population comprised consenting first ANC attendees (pregnant women) in any trimester of pregnancy. By means of the guided questionnaire via interview, age, gravidity, parity, and trimester of the first visit of participants were obtained. Pregnant women who were recruited were those diagnosed of the infections during the study period and were referred by the midwife/nurse for treatment at the health facility. Women were bled twice, at the ANC (for the RDTs) and at the laboratory for the haematological parameters (sickling, Hb, and the G6PD tests).

### 2.3. Malaria Testing

Malaria was tested on the blood samples collected from the recruited pregnant women using the SD malaria antigen P.f. (Standard Diagnostics, Inc., Korea), a rapid diagnostic test (RDT) kit. The RDT kit for *Plasmodium falciparum* (*P. falciparum*) was used since *P. falciparum* is the main *Plasmodium* species in Ghana [32–34]. The manufacturer's protocol was strictly adhered to during testing, results reading, and interpretations. This malaria RDT is sensitive to and specific for the identification of *P. falciparum* species as specified by the manufacturers. A positive result was obtained when two colour bands were observed at the test (T) line and the control (C) line in the result window. A negative result was indicated when there was only one colour band at the control (C) line. However, when no colour band was observed at both “C” and “T” columns, or at the “T” column, the tests were considered invalid and were repeated with new test kits.

### 2.4. Hepatitis B Testing

The hepatitis B infection test was conducted according to the manufacturer's instructions (Premier Co. Ltd., India, and Transnational Technologies Inc., UK) using the HBV surface antigen RDT kit, which is sensitive to and specific for the identification of the hepatitis surface antigen as specified by the manufacturers. The manufacturer's protocol was strictly adhered to during testing, results reading, and interpretations. The presence of one purple colour band at the “C” column after the incubation suggested a negative result. The presence of two purple colour bands at the “C” and “T” columns indicated a positive result. However, when no purple colour band was observed at both “C” and “T” columns, or at the “T” column, the tests were considered invalid and were repeated with new test kits.

### 2.5. HIV Testing

Before the test, a pregnant woman was taken through a pretest counselling session. After the counselling, the rapid diagnostic test, which is sensitive to and specific for the identification of HIV infection as specified by the manufacturers, was performed according to the manufacturer's instructions **(**Premier Co. Ltd., India, and Transnational Technologies Inc., UK). Results reading and interpretations of findings were done per the manufacturer's protocol. To be sure a subject was positive for HIV, a confirmatory test was performed using the OraQuick confirmation test (Premier Co. Ltd., India, and Transnational Technologies Inc., UK). The manufacturer's protocol was strictly adhered to during testing, results reading, and interpretations. In both Rapid First Response test and OraQuick test for HIV, when the result is positive, a black colour band is observed at the control line C; no colour band at C means the result is invalid and that card is faulty, and as such, the result was rejected and the test was done again with a new set of kits.

### 2.6. Syphilis Testing

The syphilis test was also conducted according to the manufacturer's instructions. The TP (*Treponema pallidum*) kit (INNOVITA (Tangshan) Biological Technology Co., Ltd., China) was used for the syphilis RDT. This RDT is sensitive to and specific for the identification of *Treponema pallidum* species as specified by the manufacturers. The manufacturer's instructions were strictly adhered to during testing, results reading, and interpretations. The development of two purple bands at both T line and C line indicated positive results. A negative result was obtained when only one purple colour band was observed at the C line. However, an invalid result was observed when no purple band appeared at the C line. In this case, the result was rejected and repeated again using a fresh test kit.

### 2.7. Haematological Testing

#### 2.7.1. Hb Estimation

Full blood count (FBC) analysis was used to estimate the Hb level among the subjects. About 5 ml of venous blood was collected into an EDTA tube. The venous blood was mixed with the anticoagulant (EDTA 2K, EDTA-3K, or EDTA-2Na) using a sample roller for a minute. The blood sample was then analyzed using the Sysmex XS-500i (Kobe, Japan). The Hb value was extracted from the FBC-printed data.

#### 2.7.2. Sickling Test

A drop of the patient's capillary blood or well-mixed venous blood was delivered on a slide. An equal volume of a fresh reducing reagent (disodium disulphite) was added, gently mixed, and covered with a cover glass. Air bubbles were excluded. A negative control and a positive control were set up alongside the sample and taken through the same treatment. After 10–20 minutes, the patient's preparation was then examined microscopically for sickle cells. Based on the outcome in relation to the negative and positive controls, the test preparation was reported as “sickle cell test positive” or “sickle cell test negative.”

#### 2.7.3. Glucose-6-phosphate Dehydrogenase (G6PD) Test

The methaemoglobin reduction test was used as described elsewhere [35]. Based on the methaemoglobin reduction test, the recruited pregnant women who were screened for the G6PD activity were classified as normal, partial, or full defect.

### 2.8. Data Analysis

Data were entered into a spreadsheet using Microsoft Excel. Then, analyses were done using Stata (version 9). Summary output tables of percentage distribution were produced for categorical variables and mean and standard deviation for continuous variables. Logistic regression analysis was used to compute odds ratios and to identify the factors significantly associated with mono- and coinfections. For all tests of association, *p* < 0.05 was considered statistically significant.

### 2.9. Ethical Clearance

Ethical clearance was obtained from the School of Medicine and Health Sciences/School of Allied Health Sciences (SMHS/SAHS) joint institutional review board (IRB), protocol number SMHS/SAHS/ER/0005. Permission was also given by the administrators of the TTH before the data were collected. Informed consent was obtained from all study subjects. However, assent was obtained from those who were below 18 years, before consent was obtained from their spouses or guardians if they were not married. All eligible subjects were provided with information about the aim and content of the study. The participants were also informed that their participation was voluntary and that they could withdraw from the study at any time without consequences and all responses received would be kept confidential.

## 3. Results

### 3.1. Seroprevalence of Malaria, Hepatitis B, HIV, and Syphilis among the Pregnant Women

During the two-year study period, 3,264 pregnant women attended ANC at Tamale Teaching Hospital, and out of these, 3,127 women consented to participate in the study and were enrolled. The remaining 137 women who declined to participate gave the following reasons for nonparticipation: lack of time to answer the questionnaire (*n*=47, 1.5%), feeling of discomfort (*n*=29, 0.9%), language barrier (*n*=16, 0.5%), and personal/undisclosed reasons (*n*=45). The age of the women included in the study ranged from 15 to 46 years with a mean of 28.5 ± 5.0 years (Table 1). About a quarter of the women (768, 24.5%) had two previous pregnancies, and a similar percentage (759, 24.4%) had no child. With respect to haematological characteristics, the mean Hb was 11.0 ± 1.4 g/dL, whilst 182 (5.8%) and 191 (6.1%) of the pregnant women had sickle cell trait and glucose-6-phosphate dehydrogenase (G6PD) deficiency, respectively. The most prevalent infection was malaria (362, 11.6%), followed by HBV (132, 4.2%). Thirty-one (31, 1.0%) of them were found to be infected with HIV, while 13 (0.4%) had syphilis infection.

Further analyses showed that, for those with monoinfections, 335 (10.7%) had malaria, 106 (3.4%) had HBV, 25 (0.8%) had HIV, and 10 (0.3%) had syphilis (Table 2). Evaluations of the interactions of infections revealed that 31 (1.0%) had a combination of any two infections (Table 3). Out of the coinfections, malaria/HBV seroprevalence was the highest (0.7%), followed by malaria/HIV (0.1%) and syphilis/HepB (0.1%). For HIV/HBV and HIV/syphilis coinfections, one woman was found in each case.

Within the 2-year period, malaria seroprevalence was revealed to be the highest (362, 11.6%), followed by hepatitis B (132, 4.2%) and HIV (31, 1.0%), with the least being syphilis (13, 0.4%) (Table 2). Malaria- and HBV-infected cases were noted every month of the study period; however, more cases were observed for malaria than HBV. On the contrary, no cases were reported in some months for HIV and syphilis infections. Within the study population, coinfection of any of the four infections under study was 31 (1.0%), with malaria-HBV coinfection being the highest (23, 0.7%) (Table 3).

To evaluate how malaria interacted with HBV and HIV, the chances of being infected with HBV and HIV were tested for those with malaria monoinfection and are indicated in Table 4. As shown in the table, infection with malaria was associated with HBV infection. This scenario was still observed when age, trimester of the first ANC visit, and gravidity were adjusted for.

### 3.2. Age and Haematological Parameters among Pregnant Women with Mono- and Coinfections

Pregnant women with syphilis were relatively younger, even though the difference was not significant (*p*=0.06) (Table 5). Mean Hb levels differed significantly among different infections (*p*=0.009). Lowest mean Hb was recorded among those with malaria and HIV, while pregnant women with syphilis recorded the highest mean Hb. Since haemoglobinopathies are known to have an effect on Hb levels of affected individuals, the distribution of some haemoglobinopathies (sickle cell trait and G6PDd) among the pregnant women was evaluated. Compared to the other infections, higher proportion of pregnant women with malaria had sickle cell trait (24, 7.2%) or G6PDd (20, 6.0%) (Table 5). Furthermore, pregnant women with HBV/syphilis coinfections were more likely to be younger, while those with HIV/syphilis coinfection were older (Table 6). The mean Hb level of pregnant women with malaria/HIV coinfection was more likely to be lower than that of those with other coinfections. Only one pregnant woman was found with G6PDd in the malaria/HIV coinfection group, whilst 2 were in the malaria/HBV group (Table 6). It was observed that no pregnant woman had more than 2 coinfections.

### 3.3. Relating Age, Trimester of the First ANC Visit, and Gravidity with Malaria, Hepatitis B, and HIV Infections among the Pregnant Women

Evaluating the relationship between age of pregnant women, time of the first ANC visit, and gravidity with malaria infection and malaria/HBV coinfection, no significant associations emerged between age, time of the first ANC visit, and gravidity with malaria infection (Table 7). However, women who for the first time attended ANC in the third trimester of their pregnancy were significantly less likely to be infected with HBV monoinfection or malaria/HBV compared to those who attended ANC in the first or second trimester even after adjusting for age and gravidity. When any coinfection was assessed, consistently, it was observed that pregnant women were significantly less likely to have a coinfection if they initiated ANC during the third or second trimester compared to the first trimester even after adjusting for age and gravidity.

## 4. Discussion

It is obvious from the study that there are interactions among malaria, HBV, HIV, and syphilis infections among pregnant women in Tamale manifesting as coinfections in some individuals. To the best of our knowledge, there are no studies in Ghana or the West African subregion that considered these four infections at the same time in pregnant women.

In this current study, malaria seroprevalence was observed every month of the year, which was consistent with the study by Owusu-Agyei et al. [36]. It was surprising to observe this appreciable number of malaria seroprevalence despite reports showing a high rollout of indoor residual spraying (IRS) programme in the three northern regions compared to the other regions in Ghana [37]. In addition, universal ownership of the insecticide-treated net (ITN) is high in the region [37, 38], but as to whether the nets are used appropriately is unknown. Thus, the results point to the fact that these interventions which were aimed at lowering malaria cases significantly within the said regions have not achieved the desired impact. Possible reasons for the high all-year-round malaria cases could include inappropriate use of the ITN and staying outdoors for long periods before getting indoors perhaps owing to the fact that rooms are usually hot especially at night. It is also possible to speculate that the high malaria cases reported all year round could be attributed to the prevalence in the general population of soil-transmitted helminth (STH) infection [39]. Although STH estimations were not carried out in this study, helminths are known to modulate host immune responses in the pregnant woman and to concurrent infections [40]. As a result, pregnant women infected with STH are immunologically compromised and are therefore highly susceptible to parasitic infections such as malaria [41].

While HIV seroprevalence and syphilis seroprevalence were relatively lower throughout the year, seroprevalence of HBV infection was high. This high prevalence of HBV infection should prompt for an intensified investigation on the history of abortion, surgery, and tattooing as these have been observed to be significantly associated with HBV infections [22]. Our study, however, did not investigate any history of abortion, surgery, and tattooing. High seroprevalence of malaria and HBV monoinfections may have accounted for the level of malaria/HBV coinfection recorded in the current study. HBV DNA load is significantly higher in coinfected pregnant women [28], which our study also confirms with the level of malaria/HBV coinfection. In the current study, the risks of malaria and malaria/HBV coinfection were lower with ANC visit initiation in the second or third trimester compared to the first trimester. As part of the ANC visits, the pregnant women are given sufficient education on preventive measures to minimise the infection rate. Late reporting for ANC is promoted by cultural practices that prohibit the reporting of pregnancies early unless some complications arise. Thus, it is possible that those who initiated ANC earlier did so because of their ill health. This can also be attributed to the cultural preferences of women in these settings to deliver at home and also not to visit ANC. Another important development in the course of HBV and malaria infections is the infective stage during their life cycle. They both invade and multiply within the liver, which might induce some stress on the liver. It was therefore not surprising that pregnant women with HBsAg were observed to have an abnormally high level of alanine aminotransferase (ALAT) [27] in HBV coinfection with another infection, suggesting liver damage.

It is found that those with malaria were significantly more likely to also be infected with HBV infection and vice versa in our study population. Only, the occurrences of the two infections were assessed, and we did not take into consideration the temporal order of occurrence of the infections and therefore cannot confirm if malaria infection increases the risk of HBV infection or vice versa. The relationship between the two infections is not clear; an increased risk of HBV infection among persons with severe malaria compared to the general public was observed in Vietnam [42], while another study reported that malaria-affected individuals with active HBV infection were more likely to be asymptomatic and to present with lower levels of parasitaemia in Brazil [16]. This finding of our study will be explored further in the future, but we invite other investigators to also explore it.

The consequences of malaria and HBV single infections result in low Hb, low CD4 count in the first trimester, low birth weight, fever, and urinary tract infection (UTI) [6, 15, 16, 21]. CD4 cell is very much important in enhancing the immune system in fighting infection via the adaptive immune system. This low CD4 count in the advent of malaria in the first trimester [21] could contribute to the increasing rate of HBV infections leading to the high malaria/HBV coinfection in the first pregnancy. Malaria and HBV single infections have detrimental impact on the health status of the pregnant woman. In view of this effect, pregnant women coinfected with malaria/HBV should be given prompt and adequate intervention so as to avoid complications.

One consequence of HIV/AIDS among others is that the immune status of these pregnant women becomes poor due to the gradual reduction in the CD4 count. A low seroprevalence rate of syphilis can be attributed to the high use of antibiotics [43] in a section of the Ghanaian population. Antibiotics are normally taken for most bacterial infections, to which syphilis belongs. Meanwhile, our study did not generate any information on antibacterial use among the participants, say in the previous one month, which could be explored in further studies. It is interesting to note that there was no malaria/syphilis coinfection among the pregnant women. Probably, a different immune mechanism and/or other factors may be at play that prevents these two infectious agents from cohabiting. On the contrary, it is noted that there are potential negative impacts on the unborn child, regarding congenital infections and mother-to-child transfer of antibodies, in relation to most of the diseases studied in this paper. These impacts include early foetal loss, stillbirth, prematurity, low birth weight, neonatal and infant death, and congenital disease among newborn babies [44–46].

Sickle cell trait status is a condition that is known to protect individuals from severe malaria. Higher percentage of sickle cell trait among single malaria infection agrees with other studies where sickle cell disease overlaps with malaria-endemic regions [47]. This percentage of sickle cell trait occurrence reduced among the pregnant women with malaria/HBV coinfection. Studies have shown that in addition to malaria disease, individuals with sickling are protected from or are associated with other diseases in terms of higher chances of being infected. The chances of HIV infection are lowered by sickle cell disease for about 70% according to an analysis of 423,431 records of adult African Americans admitted to a hospital from 1997 through 2009; it was observed that HIV infection progresses more slowly in people with sickle cell disease [48]. This same report also observed that the chances of infection with HBV or hepatitis C virus (HCV) are raised by sickle cell disease. The current study revealed that low sickle cell trait cases are observed in the pregnant women who tested positive for HIV, while high sickle cell trait cases of pregnant women were positive for HBV infection. It would have been interesting to see how sickle cell status affects the progression of HBV or HIV/AIDS. Haemoglobin genotyping would have addressed this concern, which this current study did not undertake.

The mean Hb observed in this current study was within values reported in a similar study (mean Hb of 9–15 g/dL) among pregnant women from the first to third trimester [49]. The significantly low mean Hb observed among monoinfected malaria and HIV/AIDS pregnant women signifies the impact these infections, respectively, have on the RBC. It was not surprising that malaria/HIV-coinfected pregnant women recorded the lowest mean Hb in the current study. Low Hb observed in malaria is as a result of direct lysis of the RBC due to multiplication of the *Plasmodium* parasite in RBC and also by immune mediation [50]. HIV infection on the contrary has been shown to be associated with low Hb [18, 51], and in some cases resulting in anaemic conditions [20]. HIV infection accounts for the low Hb/anaemic conditions via bone marrow suppression [52], suppression of the erythropoietin [53], and direct effect on the RBC loss [54].

G6PDd is an inherited condition caused by defect(s) in the gene that codes for the enzyme, glucose-6-phosphate dehydrogenase (G6PD). It can cause haemolytic anaemia and jaundice. The deficiency of this enzyme is quite common in areas endemic for *Plasmodium* infection [55]. Haemolytic anaemia due to G6PDd has been reported in people with HIV and hepatitis C; in hepatitis E individuals, the defect can lead to complications such as renal failure, severe anaemia, haemolysis, and hepatic encephalopathy [56]. At the ANC in TTH, sulphur- and quinine-based antimalarial drugs are used for prophylaxis, and this is a cause for worry, especially when used in pregnant women with G6PD deficiency. This may result in severe haemolysis if not monitored adequately. It is for this reason that pregnant women are screened for G6PD at their first ANC visit. Those with G6PDd are given alternative antimalarial prophylaxis and/or therapy.

Despite the significant data generated in this study, there were some limitations, which should form the basis for future studies to help strengthen the important findings here. The limitations include our inability to study the treatment and pregnancy outcomes of the study population. Furthermore, HBV profiling was not done and parasite's density was not estimated. Also, we used a cross-sectional study design which is not appropriate for studies of cause-and-effect relationships. Despite these limitations, we think the study provides some important insights into the co-occurrence of some common infections among pregnant women in Ghana.

## 5. Conclusion

Seroprevalence of 0.7% for malaria/HBV coinfection was observed in this study, where those with malaria were significantly more likely to also be infected with HBV infection and vice versa in our study population. Furthermore, risks of malaria and malaria/HBV coinfection were lower with ANC visit initiation in the second or third trimester compared to initiation in the first trimester. Our data can be relied upon to inform medical personnel, especially doctors and nurses, that any pregnant woman infected with malaria should be evaluated for HBV. While intervention on preventive health education needs to be intensified, the proportion of pregnant women with malaria/HBV coinfection calls for further studies to assess liver function among such women since malaria and HBV both adversely affect the liver.

## Figures and Tables

**Table 1:** Sociodemographic characteristics of the pregnant women who attended ANC at Tamale Teaching Hospital (*n*=3,127).

Characteristic	Frequency	Percentage (%)
*Age group (years)*		
15–19	72	2.3
20–24	622	19.9
25–29	1,143	36.6
30–34	874	28.0
35–39	354	11.3
40+	62	2.0
Total	3,127	100.0

*Gravidity*		
1	670	21.4
2	768	24.5
3	617	19.7
4	487	15.6
5	312	10.0
6	190	6.1
7	59	1.9
8+	24	0.8
Total	3,127	100.0

*Parity*		
0	760	24.4
1	759	24.4
2	614	19.7
3	461	14.8
4	297	9.5
5	162	5.2
6	45	1.4
7+	19	0.6
Total^1^	3,117	100.0

*Trimester of the first ANC visit*		
First	1,213	38.8
Second	1,051	33.6
Third	863	27.6
Total	3,127	100.0

^1^Parity has 10 missing cases.

**Table 2:** The seroprevalence of malaria, hepatitis (B), HIV, and syphilis among pregnant women who attended ANC at Tamale Teaching Hospital (*n*=3,127).

Infection	*N*	%
*Malaria*		
No	2,765	88.4
Yes	362	11.6

*Hepatitis B*		
No	2,995	95.8
Yes	132	4.2

*HIV*		
No	3,096	99.0
Yes	31	1.0

*Syphilis*		
No	3,114	99.6
Yes	13	0.4

**Table 3:** The seroprevalence of coinfections among pregnant women who attended ANC at Tamale Teaching Hospital (*n*=3,127).

Coinfection	*N*	%
*Malaria and hepatitis B*		
No	3,104	99.3
Yes	23	0.7

*Malaria and HIV*		
No	3,123	99.9
Yes	4	0.1

*Malaria and syphilis*		
No	3,127	100.0
Yes	0	0.0

*HIV and syphilis*		
No	3,126	100.0
Yes	1	0.0

*HIV and hepatitis B*		
No	3,126	100.0
Yes	1	0.0

*Syphilis and hepatitis B*		
No	3,125	99.9
Yes	2	0.1

*Any coinfection* ^*∗*^		
No	3,096	99.0
Yes	31	1.0

^*∗*^Any coinfection refers to a combination of any of the abovementioned coinfections.

**Table 4:** Associations between malaria and hepatitis B or HIV among pregnant women who attended ANC at Tamale Teaching Hospital (*n*=3,127).

	Malaria					
	Yes (*n*=362) *N* (%)	No (*n*=2,765) *N* (%)	Crude odds ratio (95% CI)	*p*	Adjusted odds ratio (95% CI)^*∗*^	*p*
*Hepatitis B*						
Yes	23 (6.3)	109 (3.9)	1.65 (1.03–2.62)	**0.034**	1.66 (1.04–2.65)	**0.031**
No	339 (93.7)	2,656 (96.1)	1.00		1.00	

*HIV*						
Yes	4 (1.1)	27 (0.9)	1.13 (0.39–3.26)	0.817	1.13 (0.39–3.28)	0.810
No	2,738 (99.0)	358 (98.9)	1.00		1.00	

^*∗*^Adjusted for age, trimester of the first ANC visit, and gravidity.

**Table 5:** Age and haematological characteristics of pregnant women with monoinfections who attended ANC at Tamale Teaching Hospital (*n*=3,127).

Characteristics	Infection type				
	Malaria	Hepatitis B	HIV	Syphilis	*p* value
*n* (%)^*∗*^	335 (10.7)	106 (3.4)	25 (0.8)	10 (0.3)	—
Mean age (SD) (years)	28.2 (5.2)	29.4 (4.4)	29.4 (5.4)	26.0 (4.4)	0.06^#^
Mean Hb^a^ (SD) (g/dL)	10.8 (1.5)	11.3 (1.4)	10.8 (1.5)	11.9 (1.4)	0.009^#^
Sickling, *n* (%)^*∗∗*^	24 (7.2)	11 (10.4)	3 (12.0)	1 (10)	—
G6PDd, *n* (%)^*∗∗*^	20 (6.0)	7 (6.6)	3 (12.0)	2 (20.0)	—

G6PDd = glucose-6-phosphate dehydrogenase deficiency. ^a^Hb was estimated in the pregnant women at the first time of ANC visit; #analysed by ANOVA. Only single infections were captured in the table. ^*∗*^Percentages were calculated based on the total study subjects of 3,127. ^*∗∗*^Percentages were calculated based on total infection for each column.

**Table 6:** Age and haematological characteristics of pregnant women with coinfection who attended ANC at Tamale Teaching Hospital (*n*=3,127).

Characteristics	Coinfection type					
	Mal + HBV	Mal + HIV	Mal + syphilis	HBV + HIV	HBV + syphilis	HIV + syphilis
*n* (%)^*∗*^	23 (0.7)	4 (0.1)	0 (0)	1 (0.0)	2 (0.1)	1 (0.0)
Mean age (SD) (years)	27.8 (3.3)	27.7 (4.8)	0 (0.0)	29.0 (0.0)	26.0 (8.4)	33 (0.0)
Mean Hb^a^ (SD) (g/dL)	11.2 (1.6)	10.4 (0.8)	0 (0)	12.4 (0.0)	11.3 (0.8)	11.0 (0.0)
Sickling, *n* (%)^*∗∗*^	2 (8.7)	2 (50.0)	0 (0)	0 (0)	0 (0)	0 (0)
G6PDd, *n* (%)^*∗∗*^	2 (8.7)	1 (25.0)	0 (0)	1 (100.0)	0 (0)	0 (0)

G6PDd = glucose-6-phosphate dehydrogenase deficiency. ^a^Hb was estimated in the pregnant women at the first time of ANC visit. ^*∗*^Percentages were calculated based on the total study subjects of 3,127. ^*∗∗*^Percentages were calculated based on total coinfection for each column.

**Table 7:** Association of malaria, hepatitis B, or malaria and hepatitis B coinfection with age, trimester of the first ANC visit, and first pregnancy among pregnant women who attended ANC at Tamale Teaching Hospital (*n*=3,127).

Infection/coinfection	Crude odds ratio (95% CI)	*p*	Adjusted odds ratio (95% CI)	*p*
*Malaria*				
Age group (years)				
** **≥20	0.98 (0.62–1.55)	0.926	0.96 (0.60–1.54)	0.910
** **<20	1.00		1.00	
Trimester of the first ANC visit				
** **First	1.00		1.00	
** **Second	1.17 (0.91–1.50)	0.235	1.16 (0.90–1.50)	0.247
** **Third	0.81 (0.61–1.07)	0.143	0.80 (0.60–1.07)	0.139
Gravidity				
** **Primigravida	0.95 (0.72–1.25)	0.738	0.96 (0.72–1.26)	0.749
** **Multigravida	1.00		1.00	

*Hepatitis B*				
Age group (years)				
** **≥20	1.33 (0.58–3.06)	0.504	1.34 (0.57–3.13)	0.503
** **<20	1.00		1.00	
Trimester of the first ANC visit				
** **First	1.00		1.00	
** **Second	0.67 (0.45–0.99)	0.046	0.68 (0.45–1.01)	0.059
** **Third	0.44 (0.27–0.71)	**0.001**	0.45 (0.28–0.73)	**0.001**
Gravidity				
** **Primigravida	1.22 (0.81–1.83)	0.340	1.20 (0.80–1.82)	0.379
** **Multigravida	1.00		1.00	

*Malaria and hepatitis B coinfection*				
Age group (years)				
** **≥20	1.40 (0.19–10.40)	0.747	1.35 (0.18–10.42)	0.773
** **<20	1.00		1.00	
Trimester of the first ANC visit				
** **First	1.00		1.00	
** **Second	0.54 (0.22–1.32)	0.175	0.55 (0.22–1.36)	0.193
** **Third	0.09 (0.01–0.67)	**0.019**	0.09 (0.01–0.68)	**0.020**
Gravidity				
** **Primigravida	1.29 (0.51–3.29)	0.594	1.23 (0.48–3.20)	0.666
** **Multigravida	1.00		1.00	

*Any coinfection of the four diseases*				
Age group (years)				
** **≥20	0.91 (0.22–3.88)	0.907	0.79 (0.18–3.46)	0.756
** **<20	1.00		1.00	
Trimester of the first ANC visit				
** **First	1.00		1.00	
** **Second	0.38 (0.16–0.90)	**0.029**	0.38 (0.16–0.90)	**0.028**
** **Third	0.19 (0.06–0.64)	**0.007**	0.19 (0.06–0.63)	**0.007**
Gravidity				
** **Primigravida	1.07 (0.46–2.49)	0.883	0.96 (0.40–2.27)	0.920
** **Multigravida	1.00		1.00	

For the adjusted models, age, trimester of the first ANC visit, and gravidity were considered; whenever the effect of one of these was being investigated, the other two were adjusted for.

## Data Availability

The datasets supporting the findings of this article are available in this manuscript.

## Abbreviations

HIV: Human immunodeficiency virus
AIDS: Acquired immunodeficiency syndrome
ALAT: Alanine aminotransferase
ANC: Antenatal clinic
CD4: Cluster of differentiation 4
G6PDd: Glucose-6-phosphate dehydrogenase deficiency
HBV: Hepatitis B virus
HBsAg: Hepatitis B surface antigen
HepB: Hepatitis B
IRS: Indoor residual spraying
ITN: Insecticide-treated nets
RDT: Rapid diagnostic test.

## Supplementary Materials

Supplementary Materials Supplementary 1. Figure 1: map of Ghana showing the study area Tamale and neighbouring communities and districts. Supplementary 2. Dataset of the subjects. This is an SPSS document containing data transcribed from the questionnaire.
